# Recurrent lung atelectasis from fibrin plugs as a very early complication of bronchial thermoplasty: a case report

**DOI:** 10.1186/s40248-015-0002-7

**Published:** 2015-03-08

**Authors:** Nicola Facciolongo, Francesco Menzella, Mirco Lusuardi, Roberto Piro, Carla Galeone, Claudia Castagnetti, Alberto Cavazza, Cristiano Carbonelli, Luigi Zucchi, Pier Paolo Salsi

**Affiliations:** Pulmonology Unit, Department of Cardiology, Thoracic and Vascular Surgery and Critical Care Medicine, Azienda Ospedaliera ASMN, Istituto di Ricovero e Cura a Carattere Scientifico, Reggio Emilia, Italy; Respiratory Rehabilitation, AUSL Reggio Emilia, S. Sebastiano Hospital, Reggio Emilia, Correggio Italy; Pathology Unit, Department of Oncology, Azienda Ospedaliera ASMN, Istituto di Ricovero e Cura a Carattere Scientifico, Reggio Emilia, Correggio Italy; Anesthesiology Unit, Azienda Ospedaliera ASMN, Istituto di Ricovero e Cura a Carattere Scientifico, Reggio Emilia, Correggio Italy

**Keywords:** Asthma, Bronchial thermoplasty, Bronchoscopy

## Abstract

**Background:**

Bronchial thermoplasty (BT) is a new therapeutic option for severe refractory asthma not controlled despite high dose inhaled corticosteroids plus long-acting bronchodilators and omalizumab in selected cases. Risk of pulmonary atelectasis after BT in severe asthma has been described in literature, but no details have been reported on the possible mechanisms of the complication.

**Case presentation:**

A 49-year-old male with severe uncontrolled asthma was referred to BT. One hour after the first procedure, acute respiratory failure occurred with PaO_2_/FiO_2_ < 300. A CT scan showed atelectasis of the right lower and middle lobes. A new bronchoscopy was performed under non-invasive ventilation; the right lower and middle lobe bronchus were occluded by bronchus-shaped plugs, that were very difficult to remove despite repeated saline washings and fragmentation with forceps. The patient had a rapid resolution of respiratory failure. Four weeks later, 6 hours after the second session of BT, severe bronchospasm occurred with respiratory failure. Chest X-Ray showed atelectasis of the left lower lobe, prompting to perform a new flexible bronchoscopy on non-invasive ventilation. The exam showed again a plug occluding the left lower lobar bronchus, removed with forceps and washings. The histological analysis of the plugs demonstrated the massive presence of fibrin with mucus debris, rare Charcot-Leyden crystals, scattered macrophages, neutrophils, eosinophils and bronchial epithelial cells.

**Conclusion:**

The originality of our case report is related to the recurrence of bronchial plugging with lobar atelectasis within one and five hours respectively, after two sequential BT procedures. At the histological evaluation the bronchial plugs appeared very different from the typical mucoid asthma plugs, being composed prevalently by fibrin. It can be hypothesized that intense thermal stimulation of the bronchial mucosa may represent a strong boost for inflammation in susceptible patients, with microvascular alteration induced directly by heat or through the release of mediators.

Although in severe asthma a risk of atelectasis from the classical asthma mucoid plugs may be expected, the peculiarity of our case resides in the formation of fibrin plugs whose direct correlation with BT should be considered.

## Background

Bronchial thermoplasty (BT) is a new therapeutic option for severe refractory asthma not controlled, despite high dose inhaled corticosteroids plus long-acting bronchodilators and omalizumab in selected cases. BT represents a really new treatment paradigm from regular daily high dose medications to a long-term and potentially permanent control of smooth muscle-induced bronchospasm [1]. The procedure is designed to target airways smooth muscle, which contributes to bronchocostriction in asthma [2]. During BT, radiofrequency energy is applied to provide thermal treatment (65°C) of visible airways > 3 mm in diameter. A special catheter, introduced via a flexible bronchoscope, is used to apply the treatment in three sessions with 3–4 week intervals. Three international randomized clinical trials in patients with asthma demonstrated the safety of this technique with an acceptable rate of post-treatment adverse events in the short-term and a significant improvement in the rate of asthma exacerbations [3-5].

Recently, results of 5-year-follow up were published with evidence of persistent AQLQ (asthma quality of life questionnaire) improvement, reduction in exacerbations, hospitalisation and emergency room accesses; good outcomes about long-term safety profile of BT were also reported [6].

The aim of this article is to report an unusual case of acute complication due to recurrent atelectasis of lower lung lobes occurring after each of 2 sequential procedures of bronchial thermoplasty.

## Case presentation

A 49-year-old male had severe bronchial asthma according to ATS/ERS definition [1]. Although the bronchodilator reversibility test was positive (FEV_1_ + 14% versus a baseline of 66% predicted), a fixed obstruction was present in stable conditions. He was a non-smoker patient, with prior nasal polypectomy, and he had common variable immunodeficiency as comorbidity. Normal ANCA levels excluded a Churg-Strauss syndrome. Asthma control was very poor (ACT score 18) with a very low quality of life (AQLQ 3.3) despite high dose inhaled therapy (budesonide 800 mcg/die, formoterol 24 mcg/die and as needed, tiotropium 18 mcg/die), and oral prednisone 8 mg daily. The use of anti-IgE monoclonal antibodies was not allowed, because serum IgE levels were above the upper safety limit for indication. The patient was referred to BT and enrolled in an experimental clinical BT-ASMN trial (ClinicalTrials.gov Identifier: NCT01839591) [7]. Standard procedures of premedication were applied, with prednisone 50 mg as required by protocol for three days, i.e. the day before, the same day, and the day after the procedure.

The first session of BT was performed on spontaneous breathing in deep sedation with remifentanil 0,10 mcg/Kg/h and propofol 8 mg/Kg/h administered under anesthesiology assistance. Copious mucus secretions and hyperemic bronchial mucosa were found at bronchoscopy examination. Fifty valid activations were administered in the bronchi of the right lower lobe without complications, such as O_2_ desaturation or bleeding.

**Figure 1:**
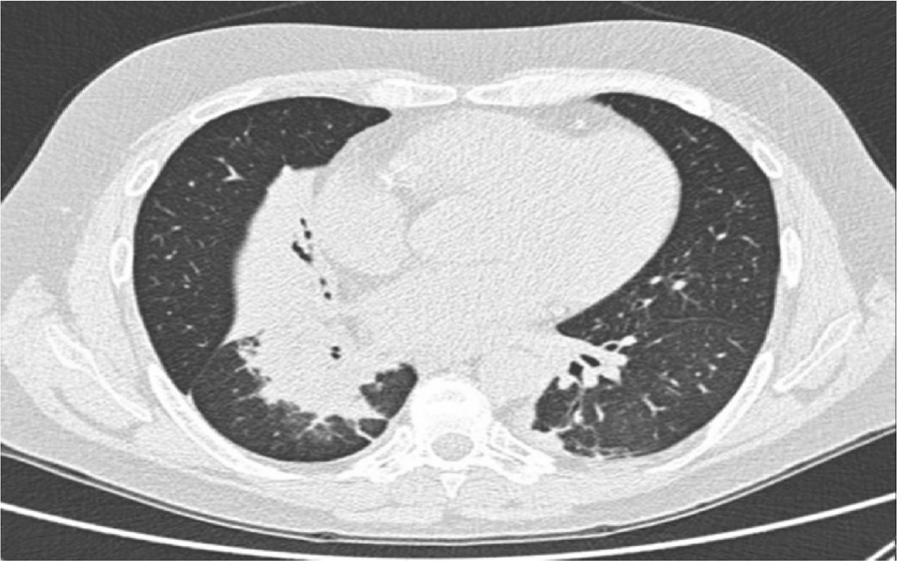
**Atelectasis of the right lower and middle lobe.**

**Figure 2:**
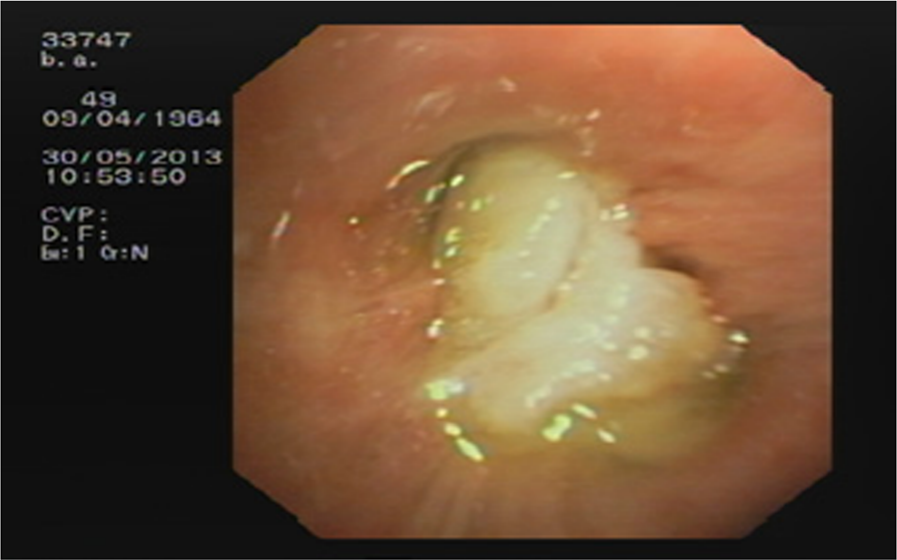
**Plugs blocking the right lower and middle lobar bronchus.**

**Figure 3:**
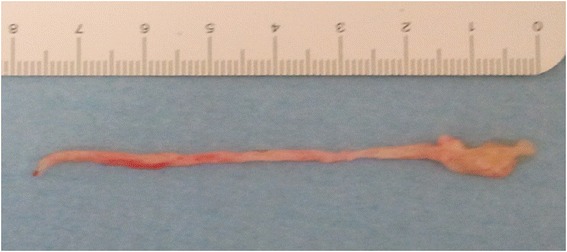
**Bronchus-shaped plug.**

After one hour, acute respiratory failure occurred with PaO_2_/FiO_2_ < 300. On physical examination, there was a reduction of breath sounds in the right lower lobe, severe bronchospasm elsewhere with tachypnea. A CT scan showed atelectasis of the right lower and middle lobes (Figure 1). A new bronchoscopy was performed on non-invasive ventilation with face mask (Esprit ventilator Philips-Respironics inc. PVC/AC mode; Performax face mask Philips-Respironics inc.) through Respironics swivel connector to ensure adequate gas exchange. Right lower and middle lobe bronchi were almost completely occluded by bronchus-shaped plugs (Figure 2). The removal of the plugs was very hard despite repeated washings with physiological saline and mechanical fragmentation with forceps (Figure 3). The patient had a rapid resolution of respiratory failure with progressive improvement of gas exchange, and was discharged after twelve days.

Four weeks later, a second session of BT was performed after the same premedication and deep sedation protocols. Sixty-one valid activations were administered in the bronchi of the left lower lobe. Bronchoscopy showed reduction in secretions as compared to the first session. Six hours later, a severe bronchospasm occurred with respiratory failure. Chest X-Ray showed a partial atelectasis of the left lower lobe. A flexible bronchoscopy on non-invasive ventilation showed again a plug occluding the left lower lobar bronchus. The plug was removed with forceps fragmentation and washings. After five days the patient was discharged.

**Figure 4:**
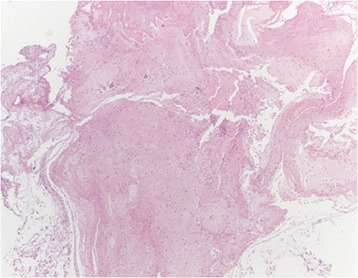
**At histologic examination, the plug consisted mainly of fibrin mixed with some mucus, scattered inflammatory cells, and some small aggregates of bronchial cells.** Haematoxylin-Eosin, 40X.

The histological analysis of the plugs demonstrated a massive presence of fibrin with mucus debris, rare Charcot-Leyden crystals, scattered macrophages, neutrophils, eosinophils and bronchial epithelial cells (Figure 4). Grocott staining was negative for microorganisms. Pan-cheratin immunostaining was negative for neoplastic cells.

The third session of BT was performed after one month. Eighty two valid activations were administered in the bronchi of the two upper lobes, without any complications.

The clinical and functional evaluation performed 12 months after the end of BT showed a good control of asthma without any kind of exacerbations. The FEV_1_ value is increased from 66% at baseline to 75% after 12 months, AQLQ score from 3.3 to 5.8 and ACT score decreased from 18 to 6.

## Discussion

Published trials have shown that BT is a safe procedure with a low incidence of adverse events, usually correlated to airways irritation [3]. The most common event is represented by exacerbation of asthma (52% versus 39% of the control group), which mainly occurs in the 24 hours after the procedure but resolves in most cases within one week. To minimize the risk of an exacerbation, patients areusually treated with oral steroids for three days, before , the day before, and the day after the procedure. In the RISA trial [4] Pavord I. et al. described two cases of segmental atelectasis secondary to treatment with BT, including one treated with bronchoscopy and another one with chest physiotherapy. In the AIR 2 [5] study, three segmental atelectasis were reported in two patients, but treatment modalities and characteristics of plugs were not described in detail. As compared to literature, the originality of our report is related to the recurrence of lobar atelectasis complication in the same patient within one and five hours respectively, after two sequential BT procedures. In addition, we report details on the histological evaluation of the mucus plugs which appear different from the typical bronchial asthma plugs.

Plugging of small and medium-sized airways by mucus and cellular debris is a well known feature of fatal asthma found in autopsy studies in both adults and children. The biochemical composition of the typical asthma plugs include plasma proteins, DNA, and proteoglycans, with mucins being the major gel-forming component. Mucins have notably different cross-linking, size, acidity and appearance (assessed by electron microscopy) compared with control mucus. Cellularity in the plugs is mainly represented by scattered eosinophils and some epithelial cells [8-11].

Helper T-cell type 2 (Th2) cytokines, including interleukin (IL)-13, are implicated in mucus production and goblet cell hyperplasia in asthma and IL-13 induces goblet cell hyperplasia with mucus hypersecretion in human airway epithelial cells [12]. Despite our patient presented with diffuse bronchial hypersecretion at the first endoscopic evaluation, as typically found in severe asthma, the composition of the post-BT plug was atypical, prevalently derived from blood serum components. It is well known that plasma exudation from the bronchial microvasculature and tissue oedema are characteristic features of asthma [13]. Vascular leakage of plasma results from inflammatory mediator-induced separation of endothelial cells in the postcapillary venules of the tracheobronchial circulation, with increase in macromolecular permeability. Plasma exudate in the airway lumen in asthma may contribute to sloughing of epithelium, impairment of mucociliary transport, narrowing of small airways, and mucus plug formation [14].

It can be hypothesized that intense thermal stimulation of the bronchial mucosa may represent a strong boost for inflammation, in susceptible patients, with microvascular alteration induced directly by heat or through the release of mediators causing mucosal exudation, oedema and formation of plugs rich in fibrin composition, as in our case. There is no explanation at the moment on the occurrence of such a mechanism only in some asthma patients and not in others. A further anomaly was represented by the fact that in the same patient the third BT procedure applied to the upper lobes did not cause atelectasis; there are no data to explain such a discrepancy between lower and upper lobes, e.g. in terms of different anatomy or physiology or factors directly related to BT.

## Conclusions

In literature**,** the number of patients treated with BT is still small to draw any definitive conclusion. It will be necessary to foster a close surveillance and reporting of adverse events correlated with BT in order to better understand how this method impacts with the anatomy and physiology of the bronchial mucosa.

## Consent

Written informed consent was obtained from the patient for publication of this case report and any accompanying images. A copy of the written consent is available for review by the Editor of this journal.

## References

[1] Chung KF, Wenzel SE, Brozek JL B, Bush A, Castro M, Sterk PJ (2014). International ERS/ATS guidelines on definition, evaluation and treatment of severe asthma. Eur Respir J.

[2] Danek CJ, Lombard CM, Dungworth DL, Cox PG, Miller JD, Biggs MJ (2004). Reduction in airway hyperresponsiveness to methacholine by the application of RF energy in dogs. J Appl Physiol.

[3] Cox G, Thomson NC, Rubin AS, Niven RM, Corris PA, AIR Trial Study Group (2007). Asthma control during the year after bronchial thermoplasty. N Engl J Med.

[4] Pavord ID, Cox G, Thomson NC, Rubin AS, Corris PA, Niven RM (2007). Safety and efficacy of bronchial thermoplasty in symptomatic, severe asthma. Am J Respir Crit Care Med.

[5] Castro M, Rubin AS, Laviolette M, Fiterman J, De Andrade LM, Shah PL, AIR2 Trial Study Group (2010). Effectiveness and safety of bronchial thermoplasty in the treatment of severe asthma: a multicenter, randomized, double-blind, sham-controlled clinical trial. Am J Respir Crit Care Med.

[6] Pavord ID, Thomson NC, Niven RM, Corris PA, Chung KF, Research in Severe Asthma Trial Study Group (2013). Safety of bronchial thermoplasty in patients with severe refractory asthma. Ann Allergy Asthma Immunol.

[7] Bronchial Thermoplasty: Effect on Neuronal and Chemosensitive Component of the Bronchial Mucosa (BT-ASMN) ClinicalTrials.gov Identifier: NCT01839591.

[8] Rogers DF (2004). Airway mucus hypersecretion in asthma: an undervalued pathology? Current opinion in pharmacology. Curr Opin Pharmacol.

[9] Sidebotham HJ, Roche WR (2003). Asthma deaths; persistent and preventable mortality. Histopathology.

[10] Sheehan JK, Richardson PS, Fung DC, Howard M, Thornton DJ (1995). Analysis of respiratory mucus glycoproteins in asthma: a detailed study from a patient who died in status asthmaticus. Am J Respir Cell Mol Biol.

[11] Dunnill MS (1960). The pathology of asthma, with special reference to changes in the bronchial mucosa. J Clin Pathol.

[12] Rubin BK (2014). Secretion properties, clearance, and therapy in airway disease. Transl Respir Med.

[13] Rogers DF, Evans TW (1992). Plasma exudation and oedema in asthma. Br Med Bull.

[14] Persson CG (1988). Plasma exudation and asthma. Lung.

